# Assessing Personality Traits in Transfemales: A Comparative Analysis with Gender-Affirming Males and Females Using a Binary Logistic Regression Model

**DOI:** 10.1192/j.eurpsy.2025.2415

**Published:** 2025-08-26

**Authors:** N. Konstantinovs

**Affiliations:** 1 First Medical Faculty, Charles University, Prague, Czech Republic

## Abstract

**Introduction:**

Transgender healthcare has increasingly gained importance, particularly in understanding how individuals align with their affirmed gender in various physiological and sociological aspects. Transfemales, individuals assigned male at birth who transition to female, represent a group that is less studied in gender research. There remains uncertainty regarding the degree of similarity transfemales exhibit to cisgender males or females post-transition. This study aims to address this by using a binary logistic regression model to compare transfemales with gender-affirming males and females.

**Objectives:**

The primary objective of this study is to determine whether transfemales are more similar to gender-affirming males or gender-affirming females. Using a binary logistic regression model previously validated on a cohort of transmales, we aim to categorize transfemales based on personality traits.

**Methods:**

A binary logistic regression model, initially developed to distinguish between gender-affirming males and females, was applied to a dataset that included transfemales. The model was trained using characteristics of cisgender males, females and transgender participants. The dataset included 108 gender-affirming males, 260 gender-affirming females, 142 transmales and 20 transfemales, ages 15-25, from Europe. The primary outcome was the classification of transfemales into the male or female categories according to personality trait assessment, using the regression function trained on the gender-affirming cohort.

**Results:**

The binary logistic regression model categorized the vast majority of transfemales as females, with a classification accuracy close to that of gender-affirming females being categorized as female. Specifically, **85%** of transfemales were classified as female, which is slightly lower than the **93%** classification accuracy for gender-affirming females but significantly higher than the **34%** classification accuracy for gender-affirming males being categorized as female. Detailed results are presented in the attached table.

**Table:** 

Group	Number of Participants	Classified as Female (%)
Gender-Affirming Males	37	34%
Gender-Affirming Females	242	93%
Transfemales	20	85%

**Conclusions:**

The results suggest that transfemales are more similar to gender-affirming females than to gender-affirming males in their personality traits based on the binary logistic regression model. However, caution must be exercised when interpreting these findings due to the limited sample size of transfemales (n = 20). Furthermore, the predictive accuracy of the model was modest, highlighting the need for further research with larger and more diverse datasets. These findings contribute to the understanding of gender identity and its alignment with personality traits, yet emphasize the complexity of such classifications in transgender populations.

**Disclosure of Interest:**

None Declared

